# Pericardial Effusion and Cardiac Tamponade in Preterm Neonates After Umbilical Venous Catheter Insertion: Management of a Case Series Using a Point-of-Care Ultrasonography (POCUS)-Guided Clinical Protocol

**DOI:** 10.7759/cureus.99821

**Published:** 2025-12-22

**Authors:** Dimitra Gialamprinou, Maria Lithoxopoulou, Eftychia Drogouti, Evgeniya Babacheva, Christos Tsakalidis

**Affiliations:** 1 2nd Department of Neonatology and Neonatal Intensive Care Unit, Papageorgiou General Hospital, School of Medicine of the Faculty of Health Sciences of Aristotle University, Thessaloniki, GRC

**Keywords:** cardiac tamponade, neonate, pericardial effusion, point-of-care ultrasound, umbilical venus catheter

## Abstract

Umbilical venous catheter (UVC) insertion is a common practice of neonatal management among neonatal intensive care units (NICUs). Although rarely reported, pericardial effusion and cardiac tamponade are potentially fatal complications of UVC insertion requiring prompt medical intervention. Echocardiography, as a point-of-care ultrasound (POCUS) practice, may enable accurate diagnosis and treatment. We report four cases of pericardial effusion after UVC insertion; two of them developed cardiac tamponade, highlighting the use of POCUS as a diagnostic modality in life-threatening complications. Ultrasound-guided pericardiocentesis was performed using echocardiography as a point-of-care applied method. Fatal UVC-related complications should be considered in neonates who suddenly develop cardiovascular instability. Echocardiography as a POCUS strategy under protocol may guide monitoring and optimal management in critically ill neonates in NICUs.

## Introduction

Central vascular catheter insertion is a standard-of-care procedure in the neonatal intensive care unit (NICU), primarily used in preterm neonates to administer hypertonic fluids, parenteral nutrition, or blood products, and to monitor hemodynamic status in critically ill neonates [1]. Despite the crucial role of central catheters in preserving life, the use of such devices is associated with mechanical, thrombotic, and infectious complications [2]. Pericardial effusion is a rare, potentially fatal complication of umbilical venous catheter (UVC) insertion when it evolves to cardiac tamponade, imposing timely intervention [3].

Premature neonates are prone to myocardial damage, mostly due to the absence of myocardium in anatomical sections of the atrial wall that is normally covered by epicardium and endocardium. The central venous catheter may cause myocardial perforation and pericardial effusion directly at the time of cannulation or secondary to line use. Given the widespread use of central catheters in the NICU setting, applying new diagnostic modalities for monitoring central catheter-associated complications has emerged as an integral part of neonatal care quality management. Echocardiography (ECHO) in the context of the point-of-care ultrasound (POCUS) is applied by trained clinicians to treat patients and has gained a principal role in everyday clinical practice in the NICU. Nowadays, POCUS may guide the monitoring of central catheters and enable effective diagnosis and management of urgent complications related to unsuitable UVC placement [4].

We report two cases of cardiac tamponade and two cases of pericardial effusion after a UVC insertion in preterm neonates, pointing out POCUS as a management tool in critically ill neonates.

## Case presentation

Case 1

A preterm female neonate was delivered in our department by Caesarean section to a 22-year-old multigravida mother at 35+6 weeks of gestational age, due to placenta previa, with a birth weight of 2,450 g. The pregnancy was otherwise uncomplicated. Immediately after birth, the neonate developed signs of respiratory distress, characterized by increasing oxygen requirements; therefore, she was intubated and received one dose of surfactant (200 mg/kg). Both a UVC and an umbilical arterial catheter (UAC) were placed for administration of total parenteral nutrition (TPN) and close monitoring of arterial blood gases, respectively.

The position of the catheters was screened by using POCUS, and a chest X-ray confirmed the position of the line tip. Initially, synchronized intermittent mandatory ventilation (SIMV) was applied until the second day of life, and thereafter, synchronized nasal intermittent positive pressure ventilation (SNIPPV) was required. At the 53rd hour of life, while the neonate was prepared for weaning from SNIPPV and was no longer requiring supplemental oxygen, she suddenly developed episodes of apnea and mild respiratory distress.

Ten hours later, multiple episodes of oxygen desaturation accompanied by bradycardia and profoundly mottled skin, cold extremities, and a capillary refill time of three seconds were observed. The neonate was intubated and was set on SIMV with low support. Sepsis screening with blood cultures was obtained, and a bolus of 0.9% sodium chloride (10 ml/kg) was administered. Due to clinical deterioration, mechanical ventilation was switched to synchronized intermittent positive pressure ventilation (SIPPV) with up-titration of respiratory support and fraction of inspired oxygen (FiO2) of 0.4.

While the neonate was mechanically ventilated, bradycardia and an increase in oxygen requirements were noted. The neonate received positive-pressure ventilation with a self-inflating bag (Ambu bag) during resuscitation. Due to the persistence of bradycardia and undetectable mean arterial pressure (MAP), thoracic compressions were applied, and adrenaline as well as inotropic support with dobutamine and dopamine were administered. The neonate was stabilized 35 minutes later.

**Figure 1:**
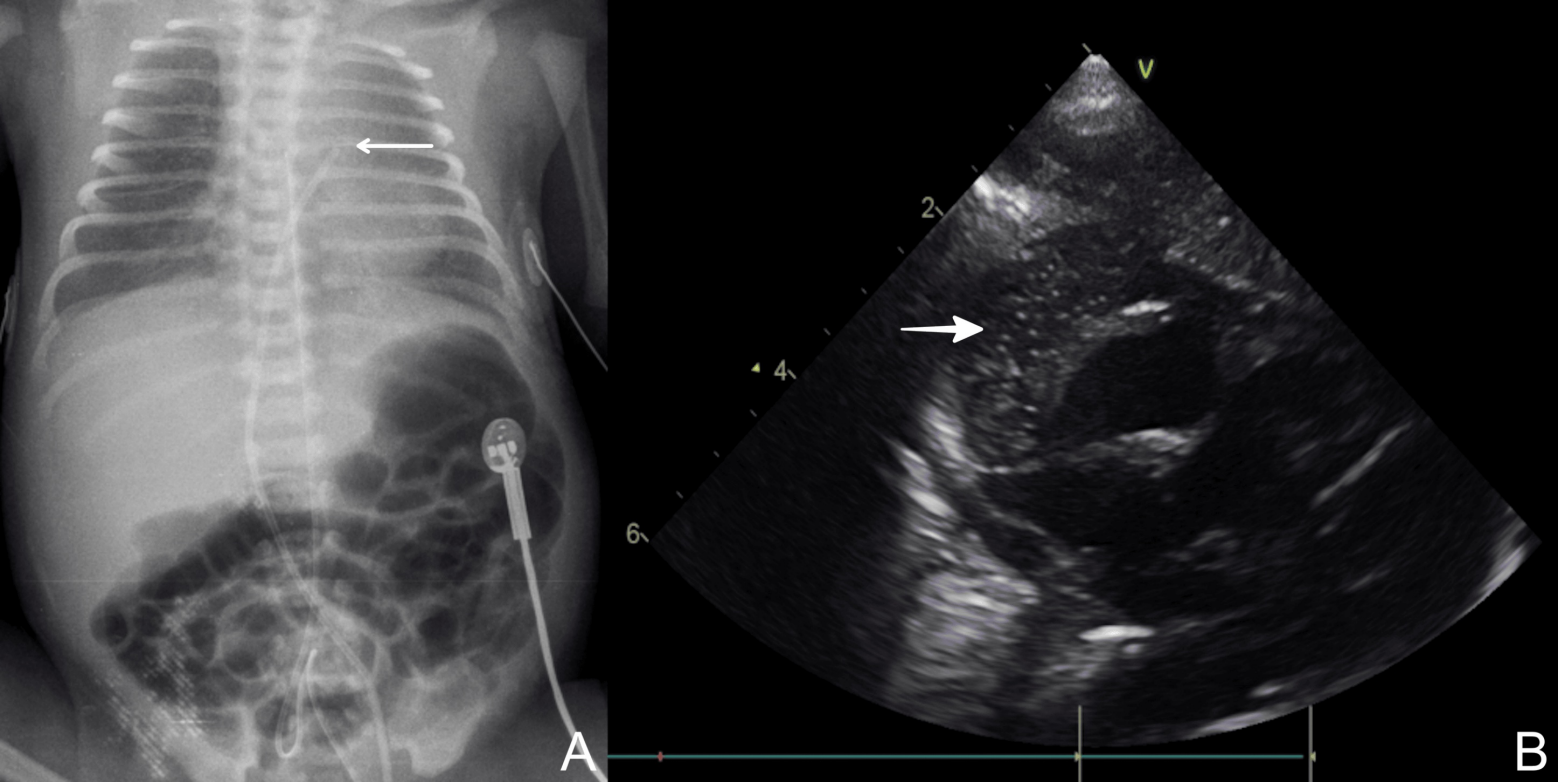
Case 1: (A) X-ray showing a high position of UVC (arrow); (B) Echocardiography showing the pericardial effusion (bold arrow). UVC: umbilical venous catheter

Chest x-ray depicted cardiomegaly and high position of the UVC, and the ECHO that followed showed the presence of pericardial effusion with evidence of hemodynamic compromise (Figure 1). Additionally, the ECHO revealed the incorrect position of UVC in the right atrial wall. The suspicion of cardiac wall injury associated with the insertion of UVC was formed, and the catheter was immediately removed. ECHO-guided pericardiocentesis was performed, resulting in drainage of 35 ml of yellow-colored fluid, which was biochemically compatible with TPN content (Figure 1B). Shortly after the intervention, the ejection fraction improved from 20% to 55%.

ECHO assessment for congenital cardiac anomalies showed a normal cardiac structure, and the culture of the extracted fluid was negative. The neonate was followed up by the cardiology team of the department for the next seven days. The pericardial effusion diminished constantly during the immediate two days after the case was managed.

During the hypoxic episode, cerebral function monitoring was applied, revealing burst suppression accompanied by clinical evidence of seizures. Anticonvulsant therapy with phenobarbital and topiramate was administered for 10 days. A mild hemorrhagic accumulation was observed in the lateral ventricles in a magnetic resonance imaging (MRI) scan four days after the hypoxic event. By 28 days of life, MRI findings were resolved. At 23 months of age, the patient remains free of clinical or laboratory evidence of brain injury, and her neurodevelopmental status is appropriate for her chronological age.

Case 2

A preterm male neonate born at 35 weeks of gestation, weighing 2,480 g, was admitted to our NICU due to prematurity and suspected early-onset sepsis. A UVC and UAC were inserted for the administration of TPN and haemodynamic monitoring, respectively. The appropriate placement of the catheters was guided by POCUS and confirmed by X-ray.

**Figure 2:**
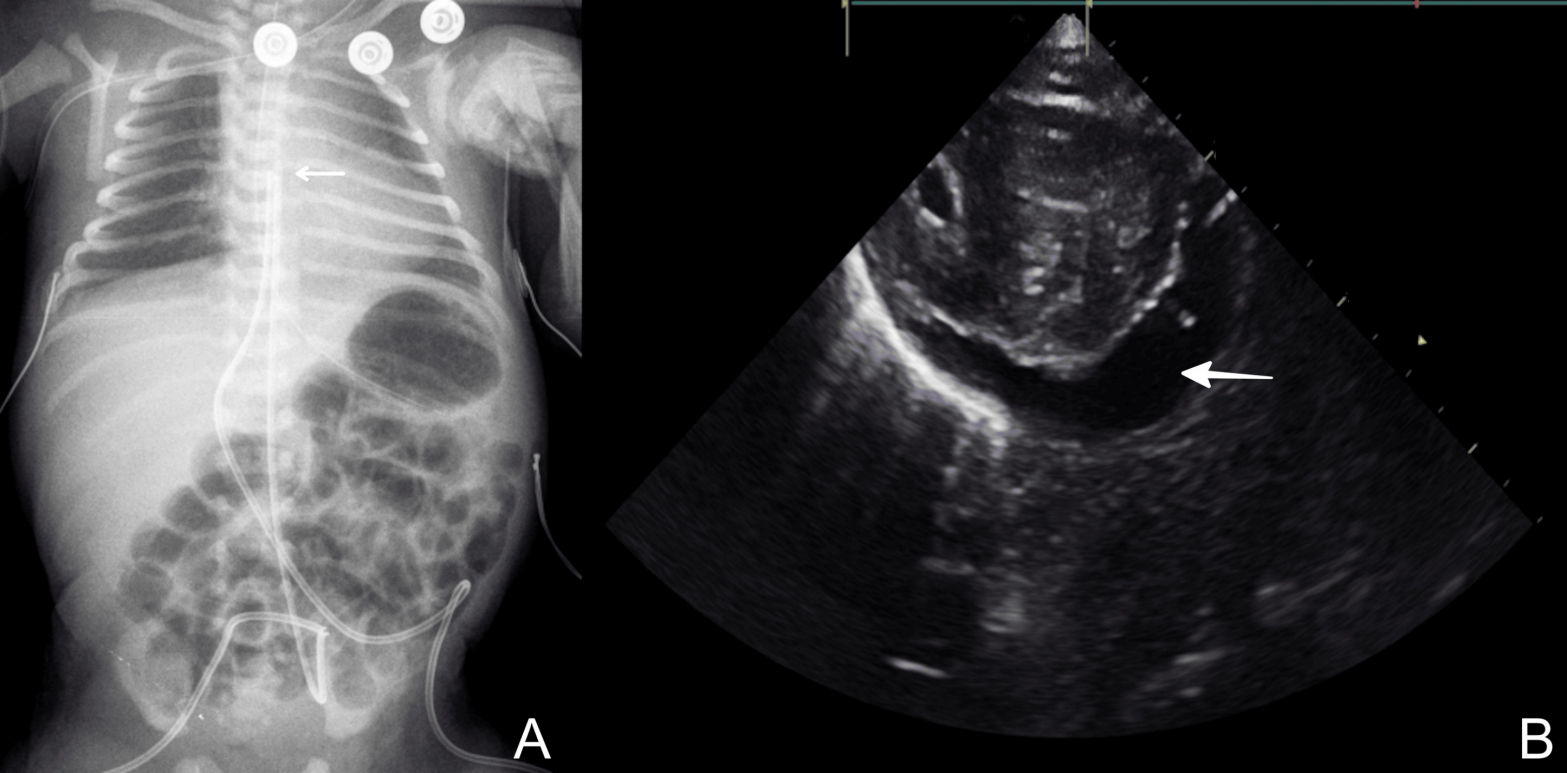
Case 2: (A) X-ray showing a high position of UVC (arrow). (B) Echocardiography showing the pericardial effusion (bold arrow). UVC: umbilical venous catheter

On the second day of life, while still receiving TPN, the infant developed hemodynamic instability, required intubation, and inotropes administration. Cardiac tamponade caused by UVC was diagnosed by POCUS (Figure 2). The patient recovered spontaneously after an emergency pericardiocentesis guided by POCUS.

Cases 3

A preterm male neonate born at 26 weeks of gestation with a birthweight of 840 was admitted to the NICU because of prematurity and RDS. The baby was intubated and set on invasive mechanical ventilation, while UVC and UAC were inserted for parenteral nutrition and antibiotics administration in the first hour of life. POCUS was used for monitoring the appropriate placement of the catheters, which was confirmed by X-ray.

**Figure 3:**
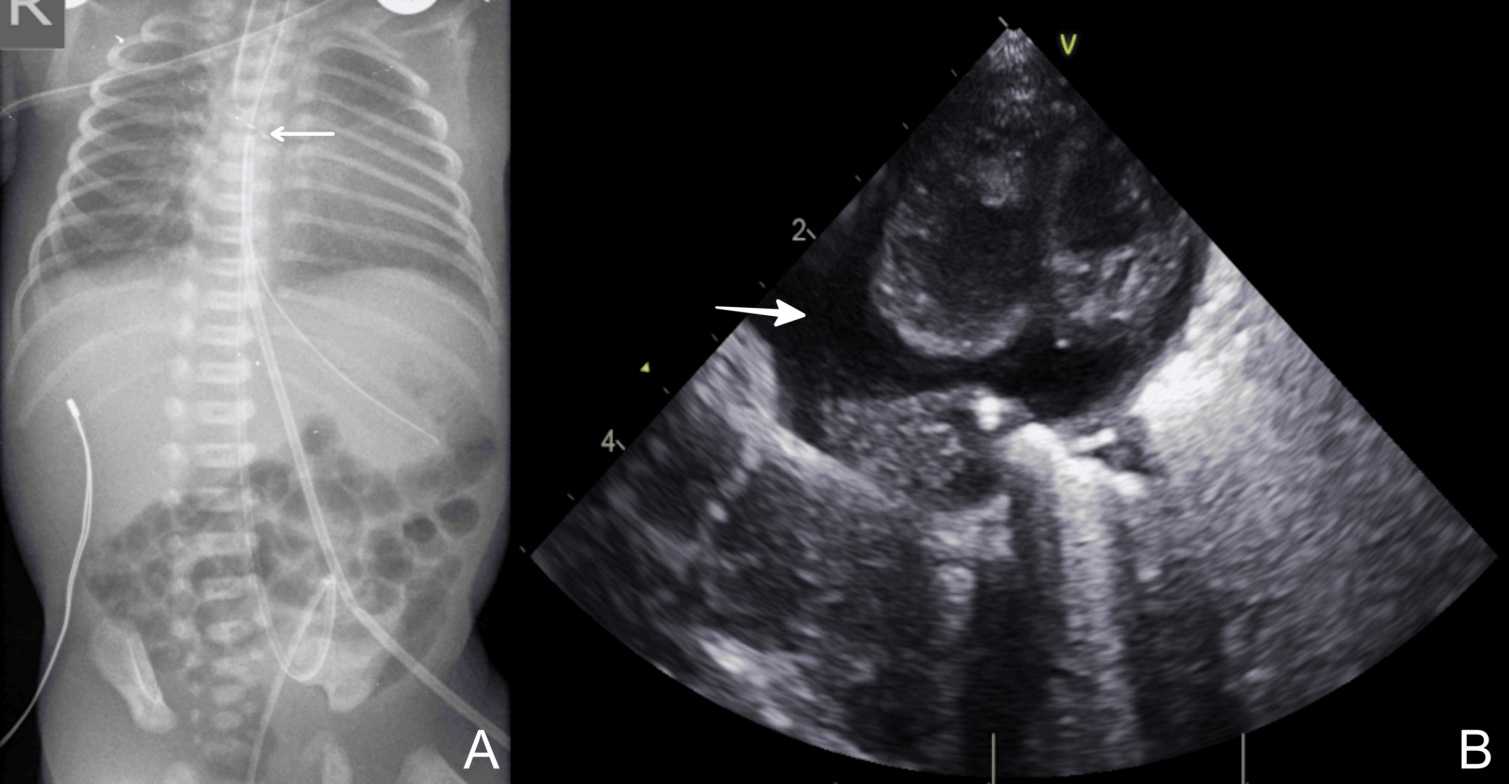
Case 3: (A) X-ray showing a high position of UVC (arrow). (B) Echocardiography showing the pericardial effusion (bold arrow). UVC: umbilical venous catheter

During the third day of life, the neonate maintained an increased need for respiratory support, and a chest X-ray was performed. The imaging revealed a wide cardiac silhouette and a high umbilical catheter position (Figure 3A). Due to the high clinical suspicion of pericardial effusion associated with the umbilical catheters’ malposition, an ECHO was performed (Figure 3B). A moderate pericardial effusion was noted; however, without leading to significant hemodynamic effects.

After consultation with a cardiology specialist, a conservative approach was decided with the appropriate replacement of the catheter. During the following two days, the neonate was under close monitoring using cardiac POCUS, and the pericardial effusion was decreased to normal measurements. The patient remained clinically stable and was weaned from mechanical ventilation without complications in the next two days.

Case 4

A preterm female neonate was born at 33 weeks’ gestation with a birth weight of 1,390 g. The infant was admitted to the NICU because of prematurity and intrauterine growth restriction and was immediately started on non-invasive mechanical ventilatory support. A umbilical venous catheter was inserted within the first hour of life, with appropriate positioning initially confirmed by POCUS and chest X-rays.

On day 3 of life, the neonate exhibited increased oxygen requirements, prompting a chest radiograph that revealed a high position of the umbilical venous catheter and an enlarged cardiac silhouette. A POCUS was urgently applied, and the malposition of the catheter tip was identified, along with a small pericardial effusion. The UVC was promptly repositioned to the correct anatomical place.

As the neonate remained hemodynamically stable, a conservative management strategy was adopted, consisting of close POCUS monitoring of the pericardial effusion. Serial echocardiography demonstrated gradual resolution of the effusion over the subsequent three days, with no associated complications.

## Discussion

In the present study, we report the cases of two preterm neonates with cardiac tamponade and two preterm neonates with pericardial effusion, all following UVC insertion. Cardiac tamponade cases were treated with pericardiocentesis, while pericardial effusion cases were managed with catheter repositioning, both followed by continuous monitoring until complete resolution with the use of POCUS.

Pericardial effusion and subsequent cardiac tamponade are rare and potentially life-threatening complications of a sub-optimal central line position, which allows leakage of infused fluids in the pericardial sac. In the literature, these complications range from 0.07% to 2%; however, mortality reaches 75% without pericardiocentesis, and decreases to below 10% when the procedure is performed [3]. A few cases of pericardial effusion and cardiac tamponade have been described in the literature. Nowlen et al. retrospectively reviewed a total of 61 cases of neonates with pericardial effusion and cardiac tamponade caused by central catheters, including 14 neonates from six hospitals in two cities of the United States and 47 cases reviewed from published data [3]. The median time from central venous line insertion to clinical presentation was three days, with a range of 0-37 days; 75% of those presented as cardiovascular collapse, while the remaining percentage showed cardiorespiratory instability of unknown causes. UVC insertion was related to 34.4% of the cases. The content of the effusion was similar to that of the infused fluid through the UVC in most of the patients. Similarly, in our cases of cardiac tamponade, the biochemical analysis of the content of the pericardial effusion points to TPN fluid, supporting the diagnosis, which was set on the third and second day of life, respectively, in line with the literature.

The recorded higher incidence of pericardial effusion in preterm neonates compared to those of term is attributed to the weakness of the myocardial wall in premature infants, which is prone to damage [3,5,6]. The contact of the catheter tip with the myocardium results in the formation of a thrombus, which increases the adhesion of the catheter tip to the myocardial wall, leading to endocardial damage and finally to pericardial effusion. Additionally, the observed sectional absence of the wall in the atrial septum, coupled with the fragility in the vessel walls in preterm neonates, fosters myocardial perforation after cannulation is performed with the UVC placement. Likewise, an osmotic injury is established after direct contact of the hyperosmolar fluid with the in direct contact with the myocardium. Interestingly, the damage to the myocardial wall may be caused directly by the irritating elements of the parenteral nutrition, even in the absence of mechanical injury to the wall [6]. Highlighting the potential life-threatening mechanical complications, monitoring and confirming the optimal positioning of the catheter, either by X-ray or ECHO, is recommended [7,8]. The tip of the UVC should be at the junction of the inferior vena cava and right atrium. In particular, the accepted position of a UVC is within 0.5 cm of the inferior vena cava and right atrium junction. The current standard for line tip positioning confirmation is still conventional thoraco-abdominal radiography, which estimates tip position in relation to the cardiac silhouette or the diaphragm [8].

Nowadays, monitoring of central line position by POCUS has emerged as an important part of neonatal care management. It has been suggested as an optimal and friendly modality for the neonatal population, ensuring security as it is free of radiation [8]. Since tip migration may frequently occur, monitoring the catheter tip over time by using ECHO is strongly recommended and has been used previously in a few studies to locate the position of central catheters in neonates. Ades et al. used POCUS to guide UVC insertion [9]. Fleming et al. used ultrasound-guided UVC insertion in neonates to confirm and monitor the location of the UVC tip [4].

**Figure 4:**
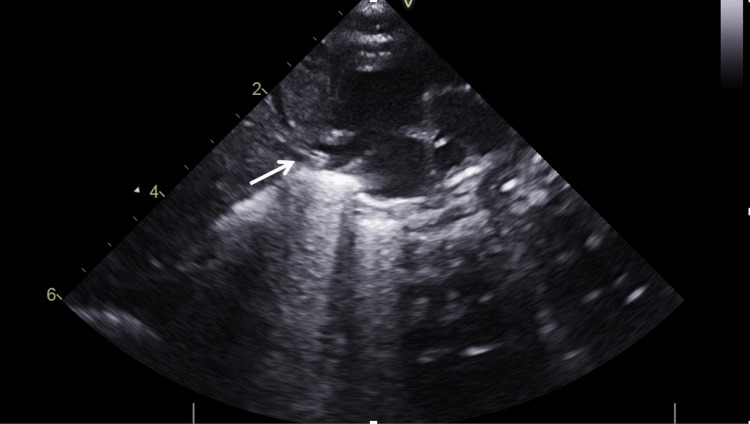
A subxiphoid ductus venous view on the right atrium for the positioning of the line tip at the junction of the right atrium with the ductus venous and the inferior vena cava

In our patients, the optimal positioning of central catheters was verified using POCUS, following a protocol that is fully integrated into our department’s daily clinical workflow. A subxiphoid ductus venous view and a left long axis parasternal view on the right atrium are obtained to ensure the positioning of the line tip at the junction of the right atrium with the ductus venous and the inferior vena cava (Figure 4). Once the optimal position of the catheter is confirmed, the line is secured by the catheter operator. A chest and abdominal X-ray is performed for each line inserted. Routine X-ray evaluation is recommended for patients with UVC tips located near the heart to verify that tip migration has not occurred. The UVC tip should be positioned outside the cardiac silhouette while remaining within the vena cava. The UVC tip should be 0.5-1.0 cm above the right diaphragm, while the UVC tip at the thoracic vertebrae 8-9 usually corresponds to the right cavoatrial junction. However, lateral and anteroposterior radiographs may overestimate or underestimate, respectively, the catheter left atrial placement.

Ultrasound has been suggested as a better modality for localization of the UVC since it more accurately confirms the position of the catheter tip and results in decreased radiation exposure than X-ray. The routine use of POCUS screening in neonates with UVC to follow any migration may early detect any complications; However, clinicians should be trained properly [8]. In our patients, the pericardial effusion and cardiac tamponade were attributed to the tip migration. In the literature, the reported definitive management of tamponade cases had been drainage of pericardial effusion [10]. In our cases, the diagnosis was confirmed, and the management of the drainage was guided by the ECHO performed in the context of the POCUS protocol.

Pericardiocentesis was performed at the 53rd and 26th hours of life when the patients deteriorated and needed cardiopulmonary resuscitation, respectively. In Case 2, there was a high index of clinical suspicion following a similar case that was recently treated by our team with the use of ECHO, and pericardiocentesis was the only intervention that proved to be life-saving for the patient, suggesting the crucial role of POCUS besides monitoring. The two neonates with pericardial effusion (Cases 3, 4) were managed by repositioning the catheter tip, followed by continuous monitoring by POCUS until complete resolution. ECHO at the bedside has been integrated as a surveillance protocol in our NICU settings to secure the optimal monitoring of hospitalized neonates.

## Conclusions

Pericardial effusion and cardiac tamponade represent rare but potentially life-threatening complications associated with the placement of UVCs. These cases require prompt identification and immediate intervention. In any neonate with an in-dwelling UVC who presents with sudden clinical deterioration, a catheter-related complication should be considered. POCUS has emerged as a safe and effective modality for verifying optimal catheter positioning and is increasingly recognised as an essential diagnostic tool in neonatal intensive care units. Current evidence suggests that POCUS is equally effective and accurate in life-threatening complications. Accordingly, future perspectives suggest that the standardized use of POCUS within a defined protocol in routine NICU practice may contribute to reducing neonatal morbidity and mortality.
